# Pitting and General Corrosion Susceptibilities of Materials for High Level Radioactive Waste (HLW) Disposal

**DOI:** 10.3390/ma15186464

**Published:** 2022-09-17

**Authors:** Brent Verhoeven, Walter Bogaerts, Pieter Van Aken, Roberto Gaggiano, Jan Baeyens, Barbara Rossi, Raf Dewil

**Affiliations:** 1 Department of Chemical Engineering, Process and Environmental Technology Lab, KU Leuven, J. De Nayerlaan 5, 2860 Sint-Katelijne-Waver, Belgium; 2 Department of Materials Engineering, KU Leuven, Kasteelpark Arenberg 44, 3001 Leuven, Belgium; 3 RD&D Department, ONDRAF/NIRAS, Kunstlaan 14, 1210 Brussels, Belgium; 4 Department of Engineering Science, University of Oxford, Parks Road, Oxford OX1 3PJ, UK; 5 Department of Civil Engineering, Materials and Structures, KU Leuven, J. De Nayerlaan 5, 2860 Sint-Katelijne-Waver, Belgium

**Keywords:** radioactive waste, disposal, deep geological formations, supercontainer, pitting, corrosion susceptibility, steel alloys

## Abstract

The disposal of high-level radioactive waste (HLW) in deep stable geological formations is accepted at an international level to be the most promising option for its long-term management. The supercontainer concept is currently being considered as the Belgian reference design, wherein the waste will be stored in geological stable clay formations. The outer barrier of the supercontainer is the envelope, which should be made of a corrosion-resistant material as it will be in contact with the aggressive species leaching from the host rock (i.e., chloride) and diffusing through the cementitious barriers of the disposal system. Polarization measurements are carried out to study the pitting susceptibility and the uniform corrosion of possible candidate materials in chloride-rich concrete pore solutions, aerated by high-purity oxygen. The tests are carried out at a deep soil-representative temperature of 60 °C. All materials showed high pitting resistance in aerated concrete pore solutions and can withstand chloride concentrations up to 1 M. Regular 316L and LDX2304 stainless steel also showed good corrosion resistance and can serve as a more economical alternative. The pH of the used pore solutions did affect the measured corrosion rate irrespective of the alloying elements inside the steel grades.

## 1. Introduction

**Figure 1:**
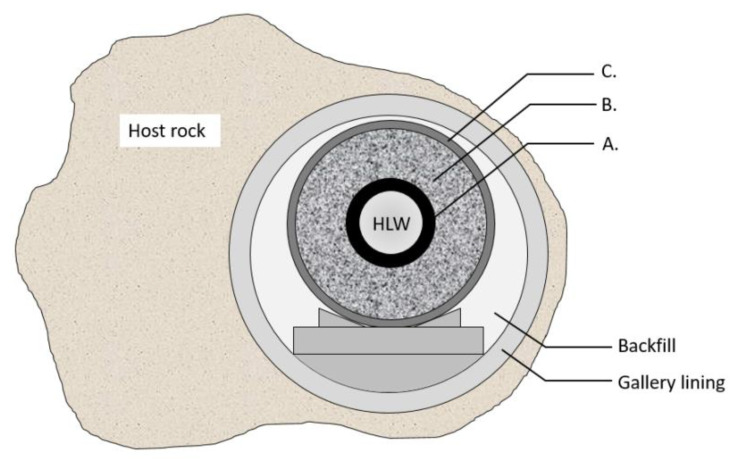
Cross-section of the “supercontainer” reference concept with its main components: (A) carbon steel liner (overpack), (B) concrete reinforcement (buffer) and (C) envelope liner.

Currently, geological disposal is internationally accepted to be the most promising option for the long-term management of high-level and/or long-lived radioactive waste (HLW). To ensure a proper sequestration of the waste, a stable geological formation is required to serve as a natural barrier. In Belgium, clay formations are favored [1,2] because of their lower water permeability, limited diffusivity and absence of preferential migration pathways for solutes. Apart from this natural barrier, the disposal system includes multiple engineered barriers: radioactive waste containers are placed in concrete shafts (galleries) in the underground facility, and the container itself (containing vitrified radioactive waste or spent fuel assemblies) consists of multiple material layers. All recommendations from the Contained Environment Concept (CEC) and even earlier designs of the Engineered Barrier System (EBS) have been addressed in the current reference design of the supercontainer (Figure 1) [3]. It consists of a carbon steel inner pack (overpack) containing the waste canister (A), surrounded by a buffer (B) and a stainless steel envelope as the outer layer (C) [4]. The intention of the CEC is to achieve and preserve a durable (long-term) favorable chemical environment in the immediate vicinity of the overpack, at least for the duration of the thermal phase (several hundred to thousands of years). Portland Cement-based concrete has hence been chosen as the material for the buffer to provide a highly alkaline chemical environment, in which the external surface of the overpack will be passivated. The main function of the envelope is preventing the infiltration of pore water from the host rock through the concrete galleries into the concrete buffer. Therefore, the envelope should have high pitting resistance, but even more importantly, a low uniform corrosion rate when it is in contact with this pore water that contains chlorides, sulfides and thiosulfates (originated from the clay host formation). Such contaminants are considerably aggressive to stainless steels and nickel-based alloys [5,6,7,8,9,10,11,12,13,14,15,16]. The concrete barriers of the disposal facility create alkaline conditions ensuring good passivation of the envelope, while elevated temperatures are expected because of the remaining heat dissipation from the nuclear waste [2]. Apart from the corrosion resistance in this harsh environment, the envelope should provide mechanical strength to ensure the reliability and possible retrievability of the supercontainer. The retrievability determines the possibility to retrieve the waste containers after they have been disposed in the repository in case the repository does not perform according to the expectations.

The concrete environment is often simulated using pore solutions in lab-scale tests. Saturated Ca(OH)_2_ (pH 12.5 at 25 °C) and a mixture of KOH/NaOH (pH 13.5 at 25 °C) are the commonly applied pore solutions seen in the literature. Li et al. [17] and Luo et al. [18] highlighted that stainless steels showed better passivation in saturated Ca(OH)_2_ than in KOH/NaOH mixtures. This is mainly due to the higher Cr^3+^ content in the passive film at pH 12.5. Regardless of the strong passivated behavior in these alkaline conditions, the presence of chlorides enhances the pitting corrosion of stainless steels.

Traditional austenitic stainless steels and lean low wt. % Ni duplex stainless steels are common alloys for applications in concrete. Several studies have reported the corrosion behavior of these alloys in simulated pore solutions. Gastaldi et. al. [19] found that AISI 304 L and LDX2101 were susceptible to pitting corrosion in saturated Ca(OH)_2_ at only 6000 ppm Cl^−^. According to Blanco et al. [20], AISI 304 L appears to show pitting susceptibility at an even lower concentration, i.e., 3000 ppm Cl^−^.

Moser et. al. [21] argued that LDX2101 has better pitting resistance than AISI 316L in saturated Ca(OH)_2_. At chloride concentrations of 0.5 M, both alloys showed pits at the surface, while LDX2304 did not show such pitting corrosion. Only at chloride concentrations of 1 M Cl^-^ did all studied lean duplex steels fail in terms of pitting corrosion [21]. Traditional duplex steels, such as DX2205, showed improved pitting corrosion resistance, even at concentrations above 1 M Cl^−^ [20,21,22]. So far, most studies performed in pore solutions have assessed corrosion behavior at ambient temperatures. It has been suggested that temperature strongly influences the pitting resistance of stainless steel. If the temperature increases, pitting potentials considerably decrease [2,19,20,21]. Pedeferri et al. [22] and Gastaldi et al. [15] performed potentiostatic and potentiodynamic measurements on stainless steels in saturated Ca(OH)_2_ at 40 °C. According to the research conducted by Gastaldi et al., austenitic stainless steels (AISI 304 L, AISI 316L) and lean duplex stainless steels (LDX2101, LDX2304) showed pitting at 40 °C with 3 wt. % Cl^−^ [15].

At the envelope surface, a temperature of 60–80 °C is expected, which is created by the heat dissipation of the enclosed radioactive waste. In addition, pitting is expected to become important because of the high chloride concentrations expected at the envelope liner, originating from the host formation. To further develop the supercontainer, and more specifically select a suitable material for the envelope, the corrosion behavior of stainless steels and nickel-based alloys should be studied in this temperature range in a chloride-rich concrete environment. Very few studies have attempted to define the corrosion behavior and the pitting susceptibility of stainless steels in pore solutions at elevated temperatures, and before now, no study has performed corrosion tests at temperatures of 60 °C. Research on corrosion behavior in chloride-contaminated pore solutions is often limited to austenitic and lean duplex stainless steels. The aim of this study is to investigate the corrosion behavior of more resistant (superaustenitic) stainless steels and nickel-based alloys in concrete pore solutions at a temperature of 60 °C. The stainless steels and nickel-based grades under scrutiny are listed in Table 1 and will be further discussed. One zirconium grade was also included, i.e., Zircaloy 702 (abbreviated as Zr702), as it shows excellent corrosion resistance in high-pH environments due to the formation of insoluble oxides [23]. Table 1 also provides the pitting resistance equivalent number, PREN, as calculated according to Equation (1).

PREN = Cr% + 3.3 * (Mo% + 0.5 * W%) + 16 * N%(1)

## 2. Materials and Methods

Accelerated corrosion tests were conducted on different stainless steels and nickel-based alloys. All tested materials with their chemical compositions and PREN values (pitting resistance equivalent number) are listed in Table 1. Every alloy is tested as a sheet under cold-rolled conditions. AISI 316L has a low PREN value and is considered as the reference material.

To simulate the concrete environment, the tests were performed in saturated Ca(OH)_2_ and a mixture of KOH/NaOH at 60 °C. The pH values of the simulated pore solutions were 12.5 and 13.5, respectively (measured at 25 °C). For testing pitting susceptibility, a high chloride concentration of 35400 ppm (1 M) was added to the pore solution. In addition, the uniform corrosion rates were studied using Tafel extrapolation [12]. All test solutions were aerated with oxygen (summarized in Table 2).

**Figure 2:**
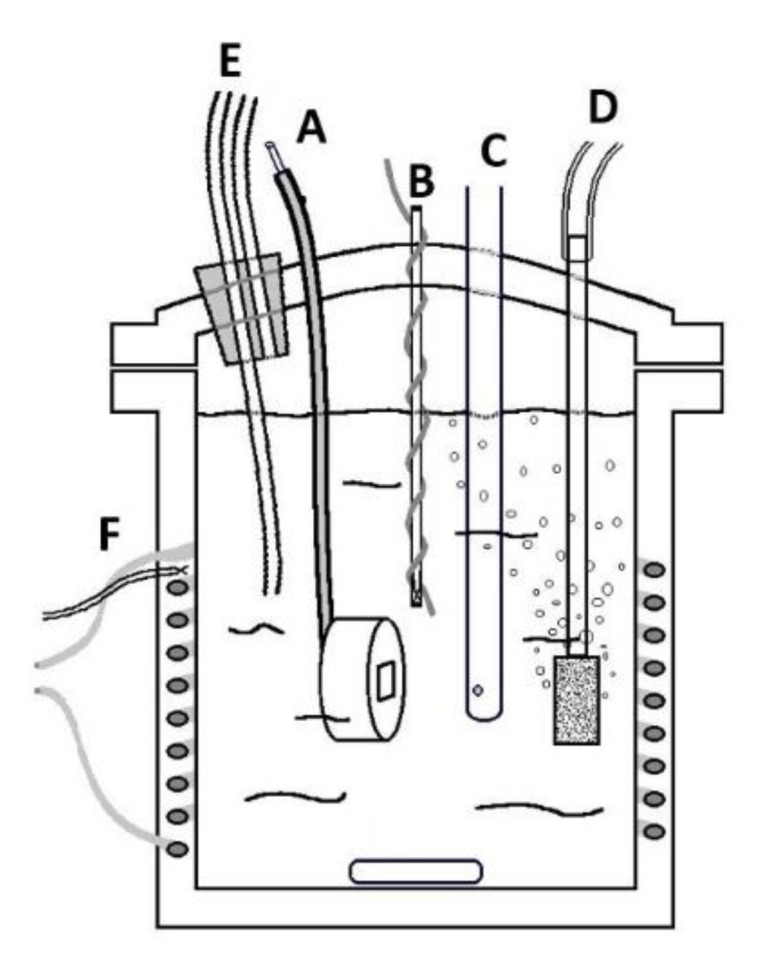
Setup of the electrochemical cell with (A) the working electrode; (B) the counter electrode; (C) the reference electrode; (D) gas bubbler; (E) gas outlet; and (F) electrical heating.

A three-electrode set-up was used to perform electrochemical corrosion tests (see Figure 2). Before each test, the sample was immersed for at least 1 h [24]. All alloys were imbedded in cold epoxy resin and polished with a diamond suspension (1 µm). A rubber gasket was compressed in the sample area to avoid crevice corrosion. A Hg/HgO reference electrode (20% KOH, +98 mV versus SHE) was used due to its electrochemical stability in alkaline solutions.

Cyclic polarization plots were used to measure the uniform corrosion rate and check the pitting susceptibility of each alloy in the test electrolyte. All polarization measurements were conducted using a scan rate of 250 mV/h. Every measurement was started 100 mV below the measured open-circuit potential. The scan direction was reversed if the apex potential was reached (+750 mV versus Hg/HgO) or if the current density exceeded 5 mA/cm^2^ [24]. Tafel extrapolation for additional information on the general corrosion behavior was conducted on the linear parts of the anodic and cathodic curves. Extrapolation should start 50–100 mV away from the open-circuit potential [12]. After checking normality with Q-Q plots, Kruskal–Wallis and Dunn test statistics were applied to get a better understanding of the data derived [25,26,27]. Additionally, a principal component analysis (PCA) provides a survey of any correlation found between the added alloying elements (within a grade) and their corresponding corrosion resistance.

## 3. Results

### 3.1. Pitting Susceptibility

**Figure 3:**
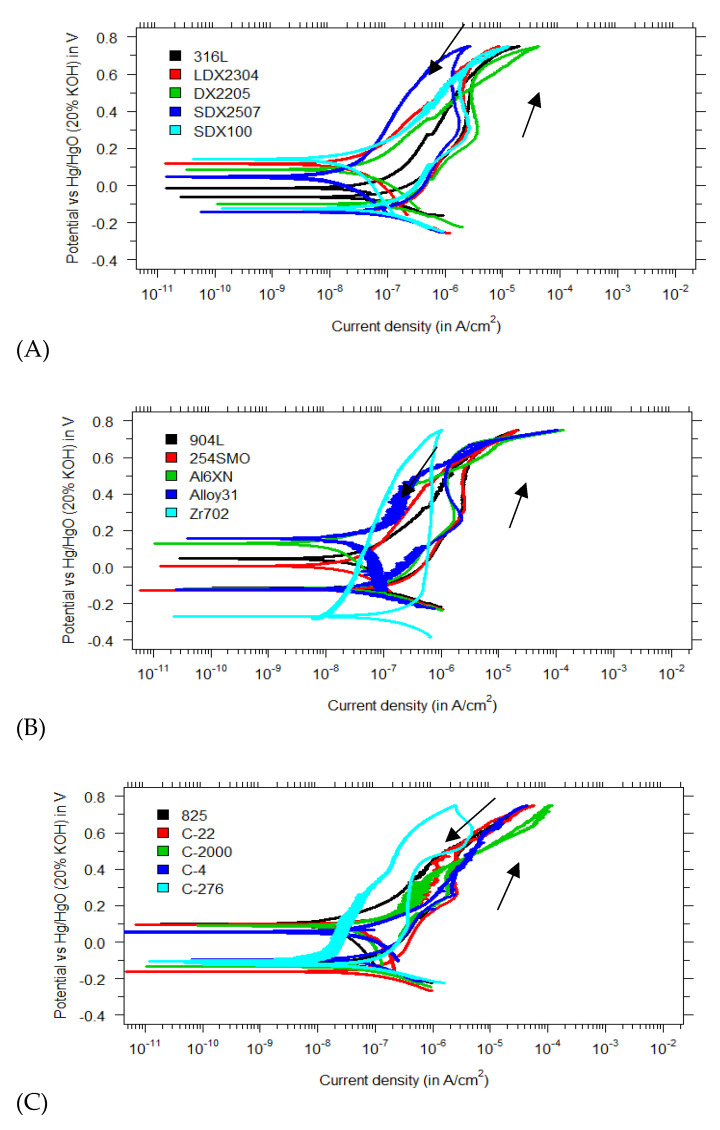
Polarization curves in saturated Ca(OH)_2_ (pH 12.5) with 1 M Cl^−^ for (**A**) regular, duplex and super duplex stainless steels; (**B**) superaustenitics and zirconium-based alloys; and (**C**) nickel—based alloys.

The results of the cyclic potentiodynamic measurements in saturated Ca(OH)_2_ with 1 M Cl^−^ at 60 °C are presented in Figure 3. Every measurement was repeated three times and showed a similar behavior. As can be seen by the absence of hysteresis loops, no pitting corrosion was found for all tested stainless steels. This result is somewhat counterintuitive, especially for the lean duplex LDX2304 and AISI 316L (reference material).

The fact that no pitting corrosion was observed for those leaner grades is in contrast with the findings of previous studies [18,20], where LDX2304 failed due to pitting corrosion at much lower chloride concentrations. A possible explanation might be that other studies were influenced by the occurrence of crevice corrosion, in which case the pitting tests performed cannot be validated. Additional uncertainty arises with the high scan rates used in other studies during potentiodynamic tests. The polarization tests of Gastaldi et al. [19] and Moser et al. [21] used high scan rates of 1200 mV/h and 3600 mV/h, respectively. ASTM standards propose a scan rate of 600 mV/h for anodic polarization curves performed on stainless steel alloys [24]. The possible interference of higher scan rates and anodic polarization curves cannot be ruled out. The exhaustive review of Esmailzadeh et al. describes the effect of the scan rate on the observed pitting potential [28]. The lower scan rates applied during anodic polarization result in lower pitting potentials. This correlation is interesting because it suggests that in the present measurements, materials are expected to fail because of the low scan rate of 250 mV/h. Another explanation for the absence of pits in the current study is the surface treatment applied. Esmailzadeh et al. suggested that polished working electrodes may relate to higher pitting resistance [28]. Ezuber found that the AISI 304 performance is significantly lower in chloride-contaminated solutions if the sample surface roughness increases [29]. In the current study, polished test specimens (up to 1 µm diamond suspension) have been used, while Moser et al. tested samples in their as-received condition [21]. Gastaldi et al. did not specify any surface treatment besides the pickling of their samples before immersing them in the test electrolyte [19].

The results for the regular duplex stainless steels are consistent with previous findings. Traditional duplex DX2205 did not show any pitting behavior in saturated Ca(OH)_2_ at 1 M Cl^−^. This is in agreement with the findings of Mesquita et al. and Elsener et al. [30,31]. More resistant materials in the experimental matrix, such as super duplex, superaustentic and nickel-based alloys, are even more promising for pitting resistance than DX2205, especially given their high PREN value (see Table 2). Bertolini et al. reported a high pitting resistance of superaustenitic 254SMO in chloride-contaminated saturated Ca(OH)_2_, even at an elevated temperature (±40 °C) [32]. The report of Nürnberger confirms that SDX100 and other superaustenitic materials (such as alloy 926) are more resistant in harsh concrete environments [33]. The results presented in this paper form one of the first investigations to explore the corrosion behavior of nickel-based alloys in simulated pore solutions. Hastelloy C-series and Alloy 825, as expected by their high PREN value, did not show any pitting susceptibility in saturated Ca(OH)_2_ with 1 M Cl^−^.

Another striking finding is the strong passive behavior of Zr702 (Zircaloy 702) in saturated Ca(OH)_2_ with 1 M Cl^−^. Unlike other polarization curves, where a current increase is observed around 200–300 mV versus the Hg/HgO electrode (probably due to dissolution of chromium as chromate), Zr702 does not show any significant current increase at high oxidizing potentials in the tested environment. This finding is consistent with the review of corrosion resistance of zirconium in ASM handbooks [34], where great performance in strong alkalis and high chloride concentrations is reported.

**Figure 4:**
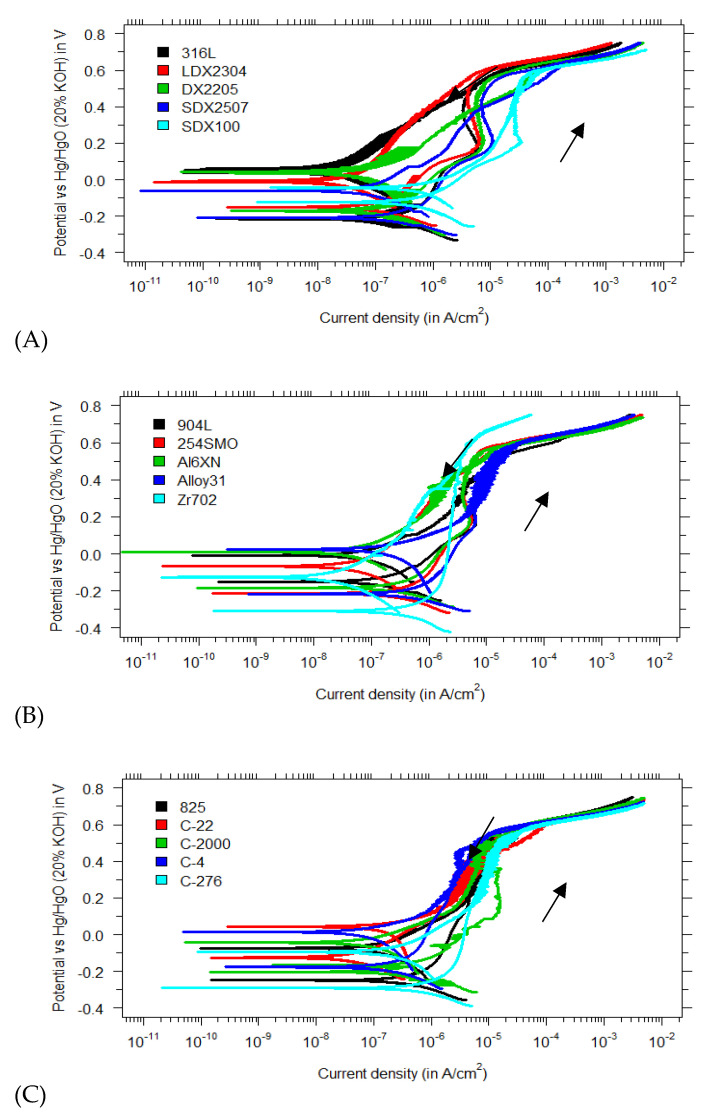
Polarization curves in a KOH/NaOH mixture (pH 13.5) with 1 M Cl^−^ for (**A**) regular, duplex and super duplex stainless steels; (**B**) superaustenitics and zirconium-based alloys; and (**C**) nickel—based alloys.

Figure 4A–C display the polarization curves of all materials in a mixture of KOH/NaOH with a high chloride content of 1 M. The absence of any hysteresis loops in the cyclic polarization curves is again remarkable. No pitting corrosion was observed on any of the test specimens. This is a significant outcome, especially given the less passive behavior of the protective film reported at pH 13.5 [17,18]. Most research on chloride-induced pitting in concrete pore solutions has been carried out in saturated Ca(OH)_2_, and the literature on higher pH is limited. Elsener et al. found that the DX2205 stainless steel did not pit in a 0.1 M NaOH solution (pH = 13), even when 5 M Cl^−^ was added [26]. Because higher pH values increase pitting resistance [35], it can be expected that duplex DX2205 will also show similar pitting resistance in KOH/NaOH pore solutions (and will also not pit in 5 M Cl^−^) [31]. No studies were found wherein other grades of stainless steel, besides DX2205, were tested in high-alkaline pore solution (pH > 13) with chloride contamination.

### 3.2. Uniform (General) Corrosion Rates

#### 3.2.1. Tafel-Extrapolated Results in Simulated Pore Solutions

**Figure 5:**
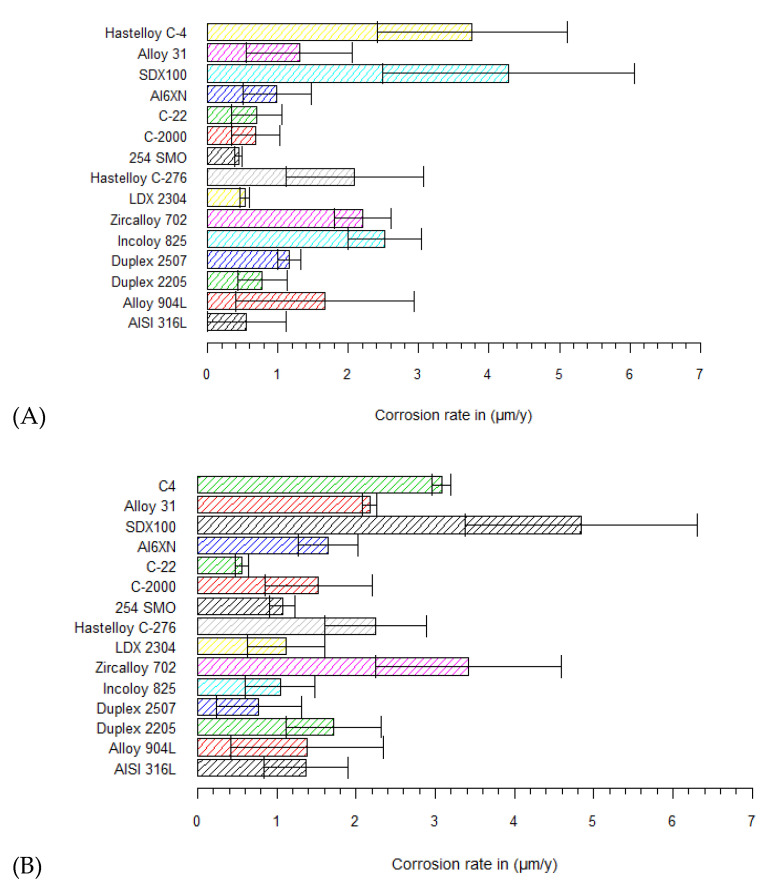
Uniform corrosion rates calculated by Tafel extrapolation in (**A**) pH 12.5 with 1 M Cl^−^; and (**B**) pH 13.5 with 1 M Cl^−^.

All corrosion rates have been determined by Tafel extrapolation. Several studies have provided insights into the importance of performing a Tafel extrapolation at least 50–100 mV away from the open-circuit potential [12]. The results in saturated Ca(OH)_2_ (pH 12.5) and KOH/NaOH mixtures (pH 13.5) with 1 M Cl^−^ (at 60 °C) are presented in Figure 5. The corrosion rates, determined by the Tafel slopes, vary between 0.44 and 4.2 µm/y.

A remarkable finding of the measured corrosion rates is the high rate of Hastelloy^®^ C-4 and SDX100 in saturated Ca(OH)_2_ (Figure 5A). Superaustenitic 254SMO shows the lowest corrosion rate, with 1 M Cl^−^. The reference material AISI 316L in saturated Ca(OH)_2_ (at 60 °C) corroded at a uniform rate of 0.56 µm/y. In the KOH/NaOH mixture (pH 13.5, Figure 5B), AISI 316L had an average measured corrosion rate of 1.46 µm/y. Again, Hastelloy C-4 and SDX100 showed high corrosion rates in pH 13.5, together with Zircaloy 702. Surprisingly, another Hastelloy grade (e.g., C-22) showed the lowest corrosion rate in pH 13.5.

Studies of stainless steels in alkaline conditions at elevated temperature with high chloride contents are limited. However, in the Cobecoma report [2], results of stainless steels in alkaline conditions have been found without chloride contamination. A corrosion rate of 0.5 µm/y was reported for stainless steels in simulated pore solutions at 80 °C. A study of Blackwood et al. has reported on experiments with AISI 304 L to check the effects of temperature on uniform corrosion rates in concrete pore solutions without any chloride addition (pH = 13) [36]. At temperatures of 50 °C and 80 °C, the corrosion rates were 0.18 and 0.82 µm/y, which are in line with the rates reported in Figure 5A. The measured corrosion rate of AISI 316L in pH 13.5 (with 1 M Cl^−^ at 60 °C~1.46 µm/y) is in the same range as the 0.6 µm/y reported by Mcdonald et al. in alkaline conditions (pH 13.3) with 0.5 M Cl^−^ at ambient temperature [37]. Yet, the outcome for the reference material 316L is different to that found by Fujisawa et al., where a corrosion rate of 0.3 nm/y was claimed for AISI 304 L in pore solution (pH 12.8). A possible explanation for this rather low rate could be the testing procedure, as Fujisawa et al. used immersion tests to determine the corrosion rate. Immersion tests are known to be more accurate and favored in determining the uniform corrosion rate [12], but often are not feasible because of their long duration.

Zircaloy 702 had a corrosion rate of 2.2 µm/y. This confirms earlier statements in the ASM Handbook suggesting a corrosion rate well below 25 µm/y in strong alkaline solutions [34]. Yau et al. found corrosion rates in alkaline conditions that were in a lower range, i.e., between 0.06 and 0.17 µm/y [38]. It is moreover important to mention that these corrosion rates were also determined by immersion tests. No studies were carried out on superaustenitic and nickel-based alloys. Other geological disposal concepts studying the corrosion behavior of Hastelloy C-series are difficult to compare because other pore–water properties were applied [39,40].

#### 3.2.2. Comparison of the Measured Corrosion Rates

**Figure 6:**
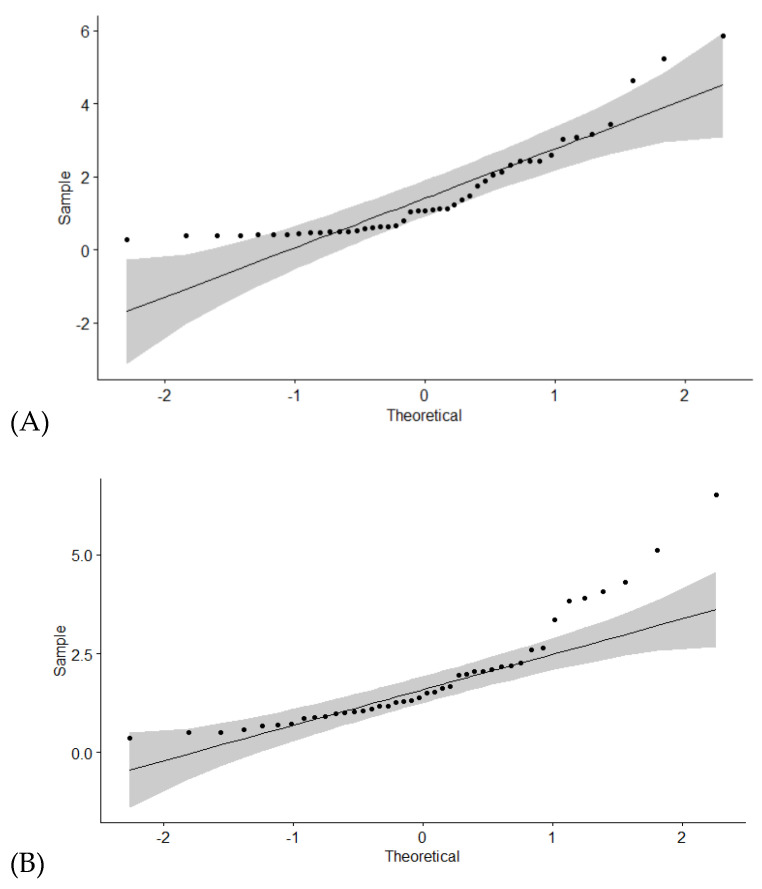
Q-Q plots used to check normal distribution of the measured uniform corrosion rates in (**A**) pH 12.5 with 1 M Cl^−^; and (**B**) pH 13.5 with 1 M Cl^−^.

Before any statistical analysis was performed, the normal distribution of the measured uniform corrosion rates was checked by a Q-Q plot (Figure 6). Theoretical quantiles of standard normal distribution have been generated (*x*-axis) and compared with the experimental quantiles of the dataset with the measured corrosion rates (*y*-axis). Points falling along a straight line give strong evidence that the dataset is normally distributed. The grey area on the Q-Q plot in Figure 6 represents a 95% confidence interval. If data points fall out of the grey area, the data are not normally distributed. In Figure 6, the Q-Q plot for uniform corrosion rates in the saturated Ca(OH)_2_ and KOH/NaOH mixture are presented. It can be concluded that, because many data points are not in the 95% confidence area for both pore solutions, the dataset is not normally distributed.

Alternatively, a Kruskal–Wallis test was performed on the corrosion data. The Kruskal–Wallis test is a non-parametric statistical test (specifically developed for not normally distributed data) and is used to check if any statistical difference is present between alloys [41]. The output of the Kruskal–Wallis test is one *p*-value for the whole dataset. A low *p*-value (<0.05) indicates statistical differences within the dataset. In our analysis, *p*-values of 0.001 and 0.025 were determined for the results in saturated Ca(OH)_2_ and a KOH/NaOH mixture, respectively. This suggests that statistical differences are present between the grades in the tested environments. A following test, the Dunn test, was used to check which alloys are statistically better than others. The results of the Dunn test are *p*-values for every possible comparison (between alloys), and if *p*-values are below 0.025, a significant difference is found.

In saturated Ca(OH)_2_ with a high chloride content (1 M Cl^−^), five materials showed different statistical *p*-values in combination with other grades according to the Dunn test (Table 3). Superaustenitic 254SMO, Hastelloy C-2000 and C-22, austenitic 316L and lean duplex 2304 stainless steel are found in several combinations with a *p*-value < 0.025. Interestingly, these five grades had a surprisingly low corrosion rate in saturated Ca(OH)_2_, as reported in Figure 5A. Superaustenitic 254SMO compared to Hastelloy C-276 had a corresponding *p*-value of 0.009, which is significant. It is apparent from Figure 5A that the uniform rate of 254SMO is lower than that of Hastelloy C-276. Even when taking into account the standard deviation, their difference is statistically significant using the Dunn test. For all the other significant *p*-values (and combinations), the same methodology was used for interpretation. 254SMO shows better resistance to uniform corrosion than Hastelloy C-4, Alloy 825, Zr702 and super duplex SDX100. The other four grades (i.e., Hastelloy C-22, Hastelloy C-2000, AISI 316L, LDX2304) showed similar behaviors to the tested 254SMO, and also have multiple comparisons with *p*-values <0.025.

The Dunn test results indicate that these five grades have a significantly low corrosion rate. The five grades with their significant comparisons are again summarized below, ranked according to their increasing (uniform) corrosion rates.

254. SMO < C-276/C-4/Alloy 825/Zr702/SDX100
Hastelloy C-2000 < C-4/SDX100
Hastelloy C-22 < C-276/904 L/Alloy 825/C-4/SDX2507/Alloy 31/SDX100
AISI 316L < C-4/Alloy 825/SDX100
LDX2304 < C-4/Alloy 825/SDX100

The same statistical test was applied to the corrosion data for pH 13.5 (mixture KOH/NaOH). Every comparison with a *p*-value < 0.025 includes super duplex SDX100, Zircaloy 702 or Hastelloy C-22. In Figure 5B, it can be observed that SDX100 and Zr702 show the highest uniform corrosion rates of all tested grades. For comparisons wherein a *p*-value < 0.025 is obtained and containing SDX100 or Zr702, the Dunn test showed a significantly lower rate for the other grades (mentioned in the comparison with SDX100 or Zr702) and, therefore, these alloys are more interesting as an envelope material in the Belgian disposal plan. The Dunn test highlights that the high corrosion rates of SDX100 and Zr702, presented in Figure 5B, are statistically significant. It is apparent (Table 4) that many comparisons are possible with Hastelloy C-22. Figure 5B illustrates that Hastelloy C-22 has the lowest corrosion rate in the KOH/NaOH mixture (pH 13.5). The Dunn test showed that the C-22 grade has a significantly lower corrosion rate than Duplex 2205, superaustenitic Al6XN, Alloy 31, Hastelloy C-4 and C-276.

Table 4 presents the outcome of the Dunn test in pH 13.5. SDX100 and Zircaloy 702 showed statistically higher corrosion rates than other tested grades. Hastelloy C-22, however, showed a promising behavior with a lower uniform corrosion rate than other candidate materials, again ranked according to increasing (uniform) corrosion rate:

SDX2507/254SMO/316L/Alloy825/Hastelloy C-22/LDX2304 < SDX100;
SDX2507/254SMO/Alloy825/Hastelloy C-22/LDX2304 < Zircaloy 702;
Hastelloy C-22 < Duplex 2205/Al6XN/Alloy 31/Hastelloy C-4/Hastelloy C-276.

**Figure 7:**
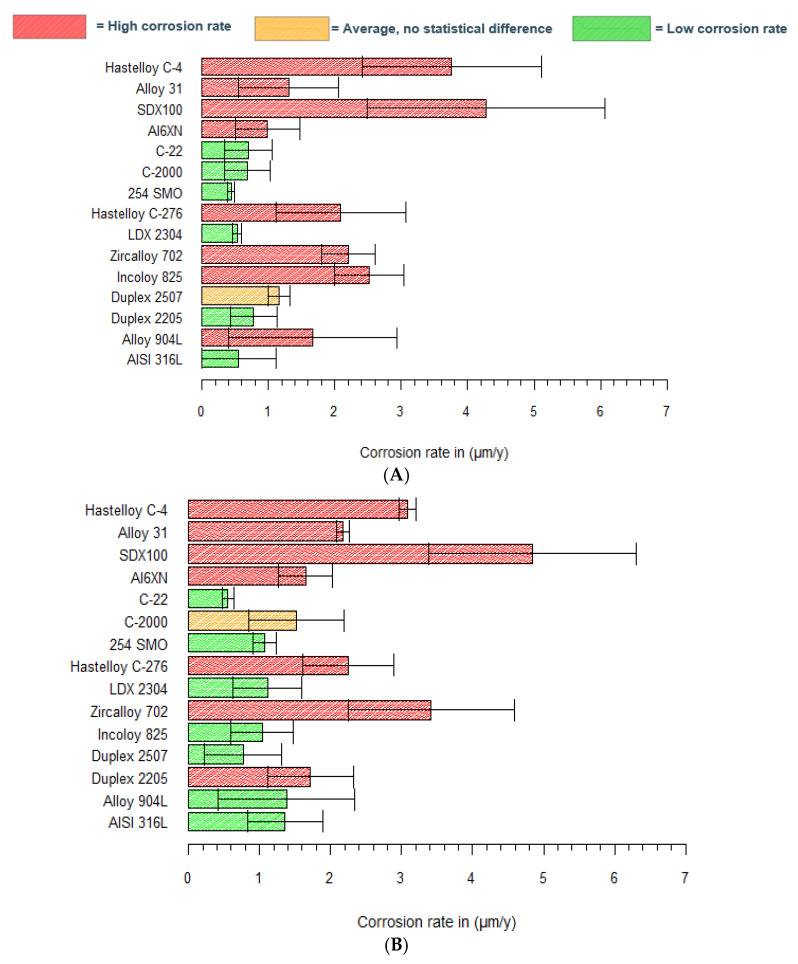
Uniform corrosion rates calculated by Tafel extrapolation, including the statistics of the Dunn tests for (**A**) pH 12.5 with 1 M Cl^−^; and (**B**) pH 13.5 with 1 M Cl^−^.

With the use of Dunn tests, significant differences between the measured uniform corrosion rates of the tested grades are identified. In pH 12.5 (saturated Ca(OH)_2_), five grades showed a significantly lower corrosion rate: 254SMO, Hastelloy C-2000, Hastelloy C-22, AISI 316L and LDX2304. Those grades are marked in green in Figure 7A, representing materials with a low corrosion rate. Hastelloy C-276, Hastelloy C-4, Alloy 825, Zr702 and SDX100 showed a significantly higher corrosion rate compared to 254SMO and, therefore, these grades are marked in red in Figure 7A. The same methodology was followed for the other comparisons. SDX2507 did not show a statistical difference with any other tested grade in the Dunn test, and is marked in orange. By combining the statistics and uniform corrosion rates, a better understanding of the performance of the tested grades in pH 12.5 is obtained. The Dunn test reveals that SDX100 and Zircaloy 702 showed a significantly higher corrosion rate in pH 13.5, and in addition, Hastelloy C-22 showed a statistically low corrosion rate. Materials that performed better than SDX100 and Zircaloy 702 are marked in green in Figure 7B. Grades that show a higher rate than Hastelloy C-22 (according to the Dunn test) are marked in red. Only one material, Hastelloy C-2000, was not found in any significant comparison, and is marked orange.

Because the envelope material should have a low uniform corrosion rate in the disposal plan, materials marked green are interesting for further consideration. Tested grades, marked in red, should be avoided because of their higher corrosion rate. Hastelloy C-22 showed a very low uniform corrosion rate in both test solutions. Implementation, however, can be limited because of its higher cost, and other grades marked in green (e.g., 316L, LDX2304 and 254SMO) can be used as a cheaper alternative.

#### 3.2.3. Effect of Alloying Elements and pH on the Measured Corrosion Rate

**Figure 8:**
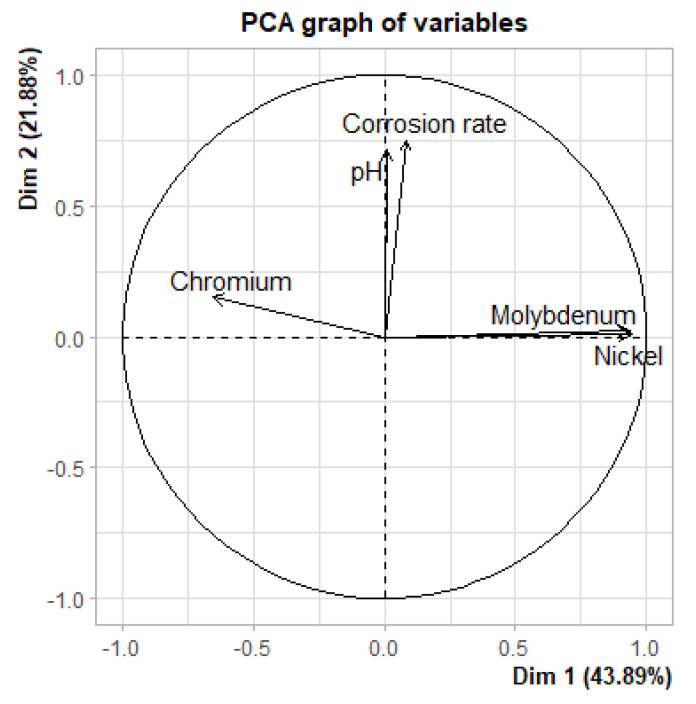
Biplot of the PCA—analysis to check the influence of alloying elements and the pH of the pore solution on the measured uniform corrosion rate.

A principal component analysis was performed to check the correlation between alloying elements, the pH of the used pore solution, and the calculated corrosion rates. This statistical procedure computes principal components and uses these to get a better visualization of the variation within the dataset. In general, two first principal components are used because, usually, they represent the largest part of the variation within the data. The values of the first principal component are mentioned on the *x*-axis, and the second component on the *y*-axis. The PCA analysis was performed in R Studio [42] and is presented in Figure 8. Alloying elements, pH and corrosion rates are represented as vectors in the biplot. If vectors have the same direction, a correlation is found. Similar directions indicate that the correlation is directly proportional. If the directions are opposed, an inversely proportional correlation is found. Vectors (and parameters) that make an angle of (almost) 90 °C on the bi-plot are not correlated. In Figure 8, the main components of stainless steel and nickel-based alloys (chromium, nickel and molybdenum) make an angle of almost 90 °C with vector corrosion rate. Therefore, little or no correlation is found between the alloying element. This result (for both pH values) is somewhat unexpected, because, especially for the chromium, a large body of the literature reports higher resistance due to increasing chromium content [43,44,45]. The vector “pH”, on the contrary, does have the same direction as the vector that represents the corrosion rate. Hence, the PCA analysis suggests a proportional correlation between both parameters. This implies that the corrosion rates measured at pH 13.5 are significantly higher than at pH 12.5. This is consistent with the studies of Li and Luo et al., who found that this adverse effect is because of a lower Cr^3+^ content inside the passive film at pH 13.5 [17,18].

To check the developed correlations of the PCA analysis, an additional Spearman test was performed (Table 5) [46]. The results of the Spearman test are in good agreement with the PCA biplot, and confirm that there is no significant effect of the alloying elements on the corrosion rate. All three alloying elements showed a high *p*-value (>0.1). Hence, the null hypothesis (no effect of tested parameter on corrosion behavior) cannot be rejected. For the pH of the pore solution, a *p*-value of 0.07 was found. A common practice is to reject the null hypothesis only if the *p*-value is lower than 0.05, although some studies suggest that 0.1 can also be used as a threshold value [47]. If a significance level of 10% (*p*-value = 0.1) is used, the null hypothesis can be rejected. By doing so, the Spearman test proves that the pH of the pore solution affects the corrosion behavior. The positive rho (ρ) coefficient suggests that the correlation between pH and the corrosion rate is proportional, similar to what was found by the PCA. However, the somewhat low value of the rho (ρ) coefficient implies that this significant effect is rather weak.

## 4. Conclusions

The aim of this paper was to study the corrosion behaviors of austenitic, superaustenitic and nickel-based alloys in contaminated pore solutions. These materials are considered as possible construction materials for the envelope of the Belgian supercontainer for the disposal of highly active radioactive waste. The study explored the pitting susceptibility and general corrosion of the promising materials under aerobic conditions.

In general, in an aerobic environment (without the presence of any reduced sulfur), good pitting resistance was observed for all tested candidate materials. No pitting corrosion was observed for LDX2304 and AISI 316L in high chloride pore solutions at 60 °C, which is somewhat surprising due to their low PREN value. Tafel extrapolation was used to calculate uniform corrosion rates. Corrosion rates of 0.44–4.2 µm/y were found in pore solutions containing 1 M Cl^−^ at an elevated temperature of 60 °C. These corrosion rates agree with other electrochemical studies, but are much higher than corrosion rates calculated by gas measuring cells (long-term immersion tests) [48]. The statistical evaluations (Kruskal–Wallis and Dunn tests) suggest that significant differences are present between the tested materials. 254SMO, C-22, AISI 316L and LDX2304 showed excellent uniform corrosion resistance in both pore solutions, and therefore, these materials are interesting for further research. However, with the higher standard deviation, caution must be applied, as comparison of Tafel extrapolated corrosion rates becomes quite difficult.

It was not possible to find a significant correlation between the main alloying elements and the measured corrosion rates. This was a surprising finding, especially because a beneficial effect of chromium on corrosion resistance is often reported in the literature [43,44,45]. The pH of the pore solution, however, did affect the corrosion behavior. This is confirmed by the PCA biplot and the Spearman test (significance level 10%). The effect was directly proportional, thus candidate materials showed a higher corrosion rate in pore solutions with higher alkalinity. A possible explanation for this may be the more protective behavior of passive layers at pH 12.5 compared to pH 13.5.

The question was raised whether lean duplex and traditional austenitic grades also perform well in more aggressive environments, as expected under sulfur-rich anaerobic conditions, which will be the topic of a follow-up paper.

## Figures and Tables

**Table 1:** Chemical composition and PREN values of all tested materials.

Common Denomination	UNS	Cr	Ni	Mo	PREN
316L	S31603	16–18	10–14	2–3	24
Alloy 825	N08825	19.5–23.5	38–46	2.5–3.5	28
904 L	N08904	19–23	23–28	4–5	32
254SMO	S31254	19.5–20.5	17.5–18.5	6–6.5	42
Al-6XN	N08367	20–22	23.5–25.5	6–7	43
Alloy 31	N08031	26–28	30–32	6–7	48
LDX2304	S32304	21.5–24.5	3–5.5	0.05–0.6	22
DX2205	S31803	21–23	4.5–6.5	2.5–3.5	31
SDX2507	S32750	24–26	6–8	3–5	38
SDX100	S32760	24–26	6–8	3–4	37
C-276	N10276	15.5–16.5	Ca. 57	15–17	-
C-4	N06455	16	Ca. 65	16	-
C-22	N06022	20–22.5	Ca. 56	12.5–14.5	-
C-2000	N06200	22–24	Ca. 67	15–17	-
Zr702	R60702	-	-	-	-

**Table 2:** Experimental conditions.

Type of Pore Solution	pH	Temperature	Chloride	Type of Aeration
Sat. Ca(OH)_2_	12.5	60 °C	35400 ppm	1 L/min O_2_
NaOH/KOH mixt.	13.5			

**Table 3:** Results of the Dunn test for saturated Ca(OH)_2_ with a high chloride content.

Compared Materials	*p*-Value of Dunn Test (*p* < 0.025)
254SMO–Hastelloy C-276	0.009
254SMO–Hastelloy C-4	0.0008
254SMO–Alloy 825	0.004
254SMO–Zr702	0.007
254SMO–SDX100	0.001
Hastelloy C-22–Hastelloy C-276	0.002
Hastelloy C-22–904L	0.01
Hastelloy C-22–Alloy 825	0.0008
Hastelloy C-22–Hastelloy C-4	0.0001
Hastelloy C-22–SDX2507	0.02
Hastelloy C-22–Alloy 31	0.015
Hastelloy C-22–Zr702	0.0014
Hastelloy C-22–SDX100	0.0002
316L–Hastelloy C-4	0.005
316L–Alloy 825	0.017
316L–SDX100	0.005
LDX2304–Hastelloy C-4	0.016
LDX2304–Alloy 825	0.004
LDX2304–SDX100	0.005
Hastelloy C-2000–Hastelloy C-4	0.007
Hastelloy C-2000–SDX100	0.009

**Table 4:** Results of the Dunn test for the KOH/NaOH mixture with high chloride content.

Compared Materials	*p*-Value of Dunn Test (*p* < 0.025)
SDX2507–SDX100	0.001
254SMO–SDX100	0.006
316L–SDX100	0.02
Alloy 825–SDX100	0.006
Hastelloy C-22–SDX100	0.0002
LDX2304–SDX100	0.007
SDX2507–Zr702	0.004
254SMO–Zr702	0.02
Alloy 825–Zr702	0.02
Hastelloy C-22–Zr702	0.0006
LDX2304–Zr702	0.02
Hastelloy C-22–Duplex 2205	0.02
Hastelloy C-22–Al6XN	0.02
Hastelloy C-22–Alloy 31	0.01
Hastelloy C-22–Hastelloy C-4	0.02
Hastelloy C-22–Hastelloy C-276	0.004

**Table 5:** A Spearman correlation test on the uniform corrosion rates.

Parameter	*p*-Value Spearman	Spearman Correlation Coefficient
Chromium	0.85	0.02
Molybdenum	0.28	0.12
Nickel	0.13	0.17
pH	0.07	0.2

## Data Availability

The data used to support the findings of this study are included within the article.
